# Sex differences in the consumption of over-the-counter analgesics among amateur volleyball players

**DOI:** 10.1186/s13102-021-00273-5

**Published:** 2021-04-29

**Authors:** Lisa Hager, Beate Averbeck, Claudia Voelcker-Rehage, Dieter F. Kutz

**Affiliations:** 1 Institute of Human Movement Science and Health, Chemnitz University of Technology, Chemnitz, Germany; 2 Walter Brendel Center of Experimental Medicine, Biomedical Center Munich, University of Munich, Munich, Germany; 3 Department of Neuromotor Behavior and Exercise, Institute of Sport and Exercise Sciences, University of Muenster, Muenster, Germany

**Keywords:** NSAID, Painkiller, Sports motivation, Volleyball

## Abstract

**Background:**

Compared with the normal adult population, athletes of several sport disciplines, such as endurance sports, ball sports, cycling and swimming, have higher use of over-the-counter analgesics (OTC analgesics). The aim of this study was to describe the epidemiology of OTC analgesic use in volleyball players as a typical competitive sport discipline. One particular focus was placed on the analysis whether the athletes’ use of OTC analgesics was influenced by their performance motivation.

**Methods:**

A cross-sectional survey among amateur volleyball players was carried out using a web-based sports questionnaire. The study included athletes of both sexes, 18 years and older, currently playing in a German volleyball league. The athletes’ sport-related complaints were evaluated regarding the use of OTC analgesics. The use of OTC analgesics by athletes was compared with their performance motivation, based on the „Achievement Motives Scale - Sport” (AMS-Sport) questionnaire.

**Results:**

The analysis of 114 completed questionnaires of amateur athletes revealed that the use of OTC analgesics was sex dependent, with a higher prevalence of use in female players (60%) versus male players (38%). The main reasons for consumption of OTC analgesics were pain in the head, knee and shoulder. The most frequently taken drug was ibuprofen, most often taken at competitions and over a period of 4 years (median). The analysis of the AMS-Sport questionnaire revealed that a logistic regression model for estimating the probability of drug use can be explained by the factors *hope of success* and *years of playing practise* in female players but not male players. In females, an increase in the factor *hope of success* resulted in a lower probability of OTC analgesic use, while an increase in *years of playing practise* resulted in a higher probability of use.

**Conclusion:**

The average duration that volleyball players in this study took OTC analgesics was higher than that of the German population, and OTC analgesic use was more prevalent in female than male volleyball players. Thus, to reduce the prevalence of OTC analgesic use, educational programs should be implemented in sports teams; and, to reduce direct and indirect social pressure, sports teams should also receive sex-specific psychological support.

**Supplementary Information:**

The online version contains supplementary material available at 10.1186/s13102-021-00273-5.

## Background

One way people balance their everyday life and improve well-being is by participating in sports in their leisure time. However, if pain occurs during sports amateur and competitive athletes use non-steroidal anti-inflammatory drugs (NSAIDs) to reduce pain and inflammation associated with training, competition or soft tissue injuries, or to gain a competitive advantage [1]. In order to suppress pain signals, amateur and competitive athletes are likely to take over-the-counter analgesics (OTC analgesics) (e.g. [2–4]). These drugs are often taken before an expected maximum exertion like a competition [3]. The use of OTC analgesics has been reported for endurance sports disciplines (i.e. [5–7]), ball sports, contact sports, cycling, swimming and mountain biking (i.e. [8–10]). The prevalence for endurance sports is between 49% [6, point measure] and 68% [7, 12 month prevalence]. Recent studies have shown that approximately 68% of cyclists take non-steroidal anti-inflammatory drugs NSAIDs [7], and at least 10% of mountain bike athletes take drugs on competition days [10].

A frequent use of NSAIDs has been shown to be associated with a 2- to 3-fold increase in gastrointestinal tract complications [11–13], cardiovascular event risks [13–15], and adverse renal events [13, 16, 17]. The incidence of adverse effects increases during or after exhausting sport competitions [5, 6], even after a short-term use of NSAIDs ([11, 13], ≤14 days; for references see: [14]). In athletes, adverse effects are also seen in fracture healing disorders, with an increased risk of non-union or delayed union and an inhibition of angiogenesis in the fracture gap [18–20]. Besides NSAIDs paracetamol is a typical OTC analgesic drug in Germany. It has the strongest pain-relieving effect in combination with ibuprofen [21] but it shows liver toxicity as a serious adverse effect [1].

Volleyball is considered as a contactless and safe sport [22, 23]. A recent comprehensive literature survey [24] names the lower limb to be the most affected area (58%) followed by upper limb injuries and injuries of the head/face/neck region (10 and 6%, respectively [24]). The risk of an injury when playing volleyball is similar to basketball and lower than other sports such as football, handball or ice hockey [25–27]. Interestingly, sex has been identified as a risk factor for some common volleyball injuries [28] such as the patellar tendinopathy “jumper’s knee” in male volleyball players and the glenohumeral subluxation in female players [29].

Athletes show a goal-oriented behaviour being based on intrinsic and extrinsic motivation [30]. Intrinsic motivation is oriented towards the athlete’s internal needs, e.g. physical performance [31] or prevention of lifestyle diseases [32], while extrinsic motivation is oriented towards the athlete’s social needs, e.g. positive stress management or social recognition [33]. Previous studies suggest that motivation is an important mediator for tolerating sport-related pain [34], and the effect of this mediator is sex dependent [34]. Performance motivation of athletes is often analysed using the questionnaire of the “Achievement Motives Scale - Sport” (AMS-Sport) [35–37]. Individual motivation is influenced by belonging to a team (i.e. [38]). Participating in a competition with pain means that an athlete runs the risk of not performing well enough and thus not being able to achieve the goals set. In this context, a “culture of risk” can be observed in the networks of athletes promoting self-harming patterns of risk, pain and injury [38–40]. This risk culture develops particularly well when the athlete networks are large enough to easily replace individual athletes. The sport networks are dense and closed in terms of the ratio of contacts within the group compared to those outside of the network. Sport networks are stable in their internal social relationship patterns, and there is a centralized flow and control over information and resources [39]. It has been shown that this concept can be applied to young elite athletes in various sports in Germany [41–43]. Recently, the concept of “culture of risk” has been further developed by introducing the risk management decision theory (RMDT, [44]) as a general framework to better understand return-to-play decision-making strategies considering decision modifiers [45]. The core concept of RMDT is a so-called “risk-defusing operator” (RDO) [44, 46–48] which is in this context an action that is anticipated to remove or reduce the risk. The decision-maker plans to carry out this action in addition to an existing alternative [48]. Here, the consumption of NSAIDs can be regarded as the RDO carried out by the player who weighs, on the one hand, the high risk of adverse effects and, on the other hand, the attractiveness to remain in the game. This extension of the concept of “culture of risk” makes it possible to interpret the behaviour of groups of non-elite athletes.

The present study aimed to describe the epidemiology of OTC analgesic use in volleyball players in Germany in a cross-sectional study using a web-based sports questionnaire. In particular, it focused on the following points. : 1.) Since pain perception differs depending on sex [49–54], and since performance motivation also mediates pain tolerance sex-dependently [34, 55], this study focused on the question whether athletes’ performance motivation is an important parameter for the athletes` use of OTC analgesics. The sport motivation was measured by means of the questionnaire AMS-Sport [37]. 2.) Since it is unknown whether and to what dose rate these drugs are taken by volleyball players in Germany we collected data about the kind of drugs taken, the quantities and frequency of OTC analgesics intake, as well as 3.) the complaints or illness (in accordance with the consensus paper of the IOC [56]) and the sporting circumstances leading to the use of drugs.

## Methods

### Sample

In this study, we conducted a cross-sectional survey of professional and amateur volleyball players of both sexes, with a minimum age of 18 years. The prevalence of self-medication over 1 y among German athletes is 21.4% [57]. This corresponds to the frequency of taking analgesics in Germany (21%, 1). To ensure that the sample contains more than 10 responders as OTC analgesic users (lower 95% confidence level), a minimum sample size of 81 participants is required. Sample characteristics are given in the results.

### Online survey

A target group-specific questionnaire in German and English was used. The survey was fully anonymous. As a consequence, the participants had no way to withdraw their consent after handing in the questionnaire. The online questionnaire was carried out using “LimeSurvey-Online-User Survey-Tool for Saxonian universities” (ver. 3.x, LimeSurvey GmbH, Hamburg, Germany). As far as possible, questions and item batteries from a questionnaire that had already validated and field-tested were used [35 items out of 44 from 37]. The part of the questionnaire on self-determined OTC analgesic use contains 44 questions, of which 35 questions were taken from the AMS Sport. The online survey was supplemented by questions about the use of OTC analgesics and then subjected to an expert assessment and a classic pilot-test in which sports students (of the Chemnitz University of Technology, Germany, *n* = 17) took part. Neither were the pilot-test data included in the analysis nor were the participants of the pilot-test asked to take part in the online survey.

The questionnaire was divided into seven sections (see Supplement):

- Introduction (explanation of the purpose of the study, privacy information and consent form)
- Medical treatment
- Use of over-the-counter analgesics (OTC analgesics) during a period of 6 months before the survey: Participants were asked to report whether they had been taking OTC analgesics, and if so, for how many years (point measurement). They had to report which of the five most common OTC analgesics in Germany (aspirin, diclofenac, ibuprofen, paracetamol and naproxen [2]) they used and which complaints (in accordance with the consensus paper of the IOC [56]) and sports situations led to the intake (period measurement of 6 months). In addition, participants were asked to report on the trade name of the drug and the frequency of intake per week (a certain fixed period of time to facilitate the participants to remember).
- Sport-psychological questionnaire from “Achievement Motives Scale – Sport” (AMS-Sport) [37]
- Demographics questions (sex, age, years of playing practise, hours of training, regularity of participation in competitions, competition class and playing position)
- End and a thank you note
- Advice concerning the risks of frequent OTC analgesic use

The “Medical treatment” set of questions served as a filter to determine participants’ current medical care. Participants taking OTC analgesics on medical advice were excluded from further analysis. Self-reported complaints that participants reported to be the reason for their OTC analgesic use were summarised and then either classified as non-sport related or categorised into 13 classes of complaints cited for sport related injuries: Headache, neck, throat, shoulder, elbow, hand, finger, back, abdomen, hip, leg, knee, foot/ankle ([25], see [58, 59]). Multiple entries were allowed. The prevalence of complaints was calculated as the total number of complaints per number of participants. The questions on OTC analgesic use relating to three different sports situations, namely competitions, training and friendly matches were answered using a 4-step bipolar Likert scale which ranged from strongly disagree to disagree to agree and strongly agree.

The AMS-Sport was processed according to the test manual published previously [37]. This questionnaire contains two motive scales: *hope for success* and *fear of failure*. Athletes who achieve a high *hope for success* score (HS, scale range: 0–45) regard performance situations to be a challenge and are convinced that they will achieve a realistic set of goals. In contrast, players with a high *fear of failure* score (FF, scale range: 0–45) are characterized by their fear of failure, as failure has to be prevented. Those athletes try to avoid competitive situations because they do not believe in success and see themselves as insufficiently prepared [37]. To interpret the AMS-Sport data, the difference of the two scores, the *net hope* = HS - FF (NH, scale range: − 45 – + 45) can be calculated. A positive *net hope* indicates that the athlete enjoys competitive (sport) performance situations whereas a negative value indicates that the athlete finds such situations unpleasant [37].

For reasons of comparability, the maximum daily dose of OTC analgesic use reported by participants was extrapolated to the average weekly dose for each individual. Demographic data encompassed age, sex, years of playing practise, hours of training, regularity of participation in competitions, competition class, and playing position. Since participants’ anonymity could be compromised by dividing the questionnaires according to regularity of participating in competitions, competition class, and playing position, these parameters were excluded from this analysis. The competition class was recoded with two values “amateur athletes” and “professional athletes”. All athletes who were traveling long distances all over Germany in order to participate in competitions and the respective tournaments were counted as professional athletes. In Germany, professional athletes playing in one of the five leagues (National team, “1. Bundesliga”, “2. Bundesliga”, “3. Liga Nord”, and “3. Liga Süd”) and take part in competitions several times a week and therefore have a higher physical load than amateur players. Following the definition of Swann and colleagues [60] these athletes have to be classified at least as semi – elite or higher while all other athletes have been classified as amateur athletes.

### Procedure

The questionnaire was accessible via a defined web link from October 1st, 2018 until March 31st, 2019. During the recruitment process, the German volleyball national associations as well as German sports institutes and universities offering a sports science curriculum were contacted by e-mail. Furthermore, a short description of the study was published on social media platforms (Facebook pages or homepages of volleyball clubs) at regular intervals. Due to technical problems in some local sports clubs, some questionnaires were made available in paper form. These questionnaires were manually transferred into the database by one of the authors (LH).

### Statistical analysis

All statistical analyses were performed using “R: A Language and Environment for Statistical Computing” (version 3.6.3, R Core Team 2020, Vienna, Austria) in combination with the GUI RStudio (version 1.2.5042, RStudio, Boston, MA). Comparisons between groups were calculated using the Wilcoxon rank-sum test and Pearson’s χ^2^ test, with false-discovery-rate adjustment where appropriate. For all statistical tests of the descriptive data, a significance level of *p* < 0.05 was determined.

The probability of OTC analgesic use was estimated using binary logistic regression analysis, identifying and quantifying the effects of predictor variables on a dichotomous dependent variable. The logistic regression was conducted with the parameters *age*, the AMC parameters *hope of success* and *fear of failure* and the sport-related parameters *years of playing practise* and *hours of training*. The regression models were estimated for each sex separately. In an oversized model, each unnecessary parameter introduces additional variability, which is distributed across all parameters. Model variability was estimated using the Akaike information criterion (AIC). Parameters were eliminated stepwise by means of the function step() in R. This algorithm is an iteratively reweighted least-squares algorithm [61] which explores the impact of adding or dropping a variable from the current model, one at a time. The function suggests a reduced model, basing the decisions on the values of the Akaike information criterion [62, 63].

## Results

### Sampling characteristics

Out of a total of 259 visits to the website, 152 questionnaires (age range 10–58 years, 100 female) were fully answered. A total of 15 questionnaires had to be excluded from the analysis: Five participants were minors, two did not play volleyball and eight were taking OTC analgesics under medical supervision at the time of the survey. As a result, a total of 137 questionnaires were available for further analysis. The data were split into professional and amateur athletes (see Methods) and further analyses was done with the data of amateur athletes’ only, as anonymised reporting of professional players was not possible due to the small number of data. In total, data from 72 women and 42 men were further analysed. On average, the female volleyball players were 26.6 ± 6.0 years old (mean ± standard deviation [SD]) and the male players were 29.3 ± 9.6 years old. Forty-two female players (58%) and 16 male players (38%) reported to take OTC analgesics without a medical prescription in the last 6 months prior to the survey. Female players reported taking OTC analgesics over a period of 5.5 ± 5.5 years and male players over a period of 4.6 ± 3.2 years.

Due to the significant difference between the two sexes of amateur athletes in terms of the proportion of reported OTC analgesic use (Pearson’s χ^2^, *p* < 0.05), the data was analysed separately for both sexes. Comparing OTC analgesic users and non-users, female players differed in age and years of playing practise but not in the hours of training per week (Student’s t-Test, *p* < 0.05). Male players differed neither in age, nor years of playing practise or training hours (Table 1). The distribution of intake frequencies separated by sex and age group is given in Table 2.

**Table 1:** Demographic data of female and male amateur volleyball players using or not using OTC analgesics

		OTC analgesics-user	non-user
Women	*N*	42^**+**^	29
	age (year) *	28.0 ± 6.3, 18–47	24.5 ± 4.9, 18–36
	years of playing practice *	14.7 ± 5.9, 4–34	11.9 ± 5.3, 3–23
	hours of training (per week)	7.4 ± 4.1, 2–20	9.2 ± 3.8, 3–20
	OTC use without medication (years)	5.5 ± 5.5, 0–20, *N* = 39	
Men	*N*	16	26
	age (year)	29.4 ± 10.7, 18–57	29.2 ± 9.1, 18–51
	years of playing practice	15.6 ± 1.2, 1–42	14.5 ± 9.7, 1–42
	hours of training (per week)	11.5 ± 7.7, 2–32	11.2 ± 5.9, 2–25
	OTC use without medication (years)	4.6 ± 3.2, 0–10 *N* = 13	

Values are presented as mean ± SD, range. * significant different between OTC analgesic-user and non-user, *p* < 0.05, ^**+**^ one female OTC analgesic-user did not report her age

**Table 2:** Proportion of reported OTC analgesic use by sex, and age group among amateur athletes

	intake	< 25	25–29	30–34	35–39	≥ 40
Women	Y	13	13	10	4	2
	N	15	11	3	1	0
Men	Y	6	5	1	1	3
	N	6	10	1	3	4

*Y*: OTC analgesics-user, *N* non-user

### Complaints

**Fig. 1:**
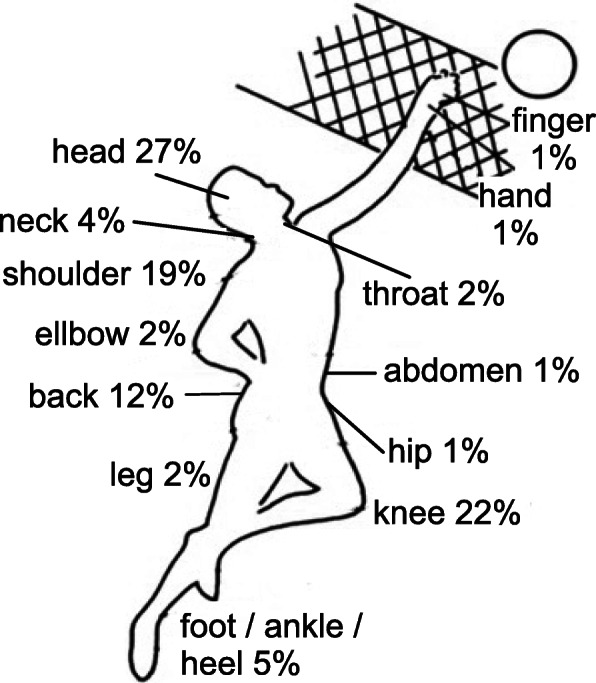
Distribution of complaints reported by amateur athletes to be the reason for the use of OTC analgesics

The players in the survey reported about 119 complaints (reasons) for taking OTC analgesics. A total of 83 complaints correspond to illness that can occur during sports activities (see Methods); the remaining 49 were everyday complaints, like fever, cold symptoms, dental surgery and menstrual problems (cited by eight female players), and thus these complains were excluded from further analysis. The distribution of the 13 different sports-related complaints found in the study is shown in Fig. 1. The most commonly mentioned complaints were pain in the head (27%), knee (22%), shoulder (19%) and back (12%). The prevalence was 1.7 times higher in female players than in male players. Nevertheless, there was no significant sex difference regarding the naming of complaints (Table 3, Pearson’s χ^2^_(DoF = 12)_ = 9.744, *p* = 0.64).

**Table 3:** Injuries mentioned by amateur athletes as the reason for taking OTC analgesics (multiple answers possible)

	Volleyball specific injuries	women	men
Upper part of the body	headache	19	3
	throat	1	1
	neck	2	1
	shoulder	11	5
	elbow	1	1
	hand	0	1
	finger	1	0
	back	9	1
	abdomen	1	0
Lower part of the body	hip	1	0
	leg	1	1
	knee	12	6
	foot / heel / ankle	3	1
	**Total**	**62**	**21**

### Use of over-the-counter analgesics

**Fig. 2:**
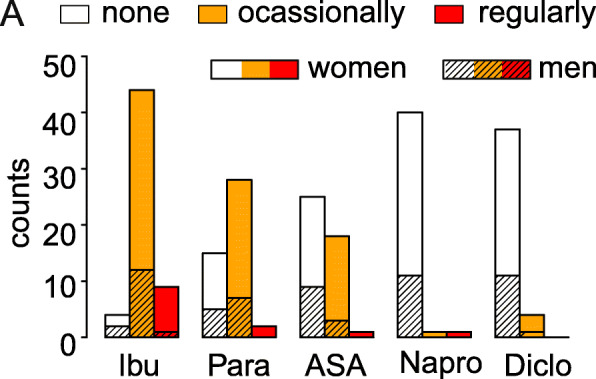
Frequency of the use of the five common OTC analgesics by amateur athletes in Germany. None: player did not consume that respective drug; filled bars: women; hatched bars: men; Ibu ibuprofen; Para = paracetamol ASA = aspirin; Napro = naproxen; Diclo = diclofenac

The most commonly taken OTC analgesics reported by the participants of this study were ibuprofen (53 players) and paracetamol (30 players). Naproxen and diclofenac were taken the least often (Fig. 2). Of the 53 players taking ibuprofen, 27 took paracetamol in addition, 17 aspirin, 3 diclofenac and 3 naproxen. Eleven players reported to take no additional analgesic drug, and 8 players gave no further information about taking additional analgesics.

Regarding the dosage of the OTC analgesics, 39 of the 114 volleyball players provided information about the name of the drugs used, the number of tablets taken, the dosage of a single tablet and the regularity of use per week. Of these 39 players, 33 reported taking OTC analgesics regularly during the week, while the other 6 reported taking the drug occasionally at irregular intervals. The most frequently taken drug was ibuprofen (*N* = 31) in the dose of 400 mg (median; inter-quartile range (IQR): 100 mg; range: 100–800 mg) as a single dose or 550 mg per week (median; IQR: 500; range: 100–2800). The self-reported duration of ibuprofen intake was in median 4 years (IQR: 6; range: 0–20) more or less continuously.

The prevalence of taking OTC analgesics depended on the sporting situation. In the survey, 57 players reported on the sporting situation (competitions, training and friendly matches) when taking OTC analgesics. Forty-five players (79%) agreed (or strongly agreed) to the statement of taking OTC analgesics during sports competitions. In contrast, the prevalence of taking OTC analgesics before training or friendly matches was significantly lower (Table 4, training: Pearson’s χ^2^_(DoF = 9)_ = 18.547, *p* = 0.029; friendly matches: Pearson’s χ^2^_(DoF = 9)_ = 16.761, *p* = 0.053).

**Table 4:** Sports situations in which amateur volleyball players use OTC analgesics

Situation	Strongly agree	Agree	Disagree	Strongly disagree
Training	3	14	24	16
Competition	19	26	8	5
Friendly matches	4	15	14	24

### Achievement motivation scale scores

**Fig. 3:**
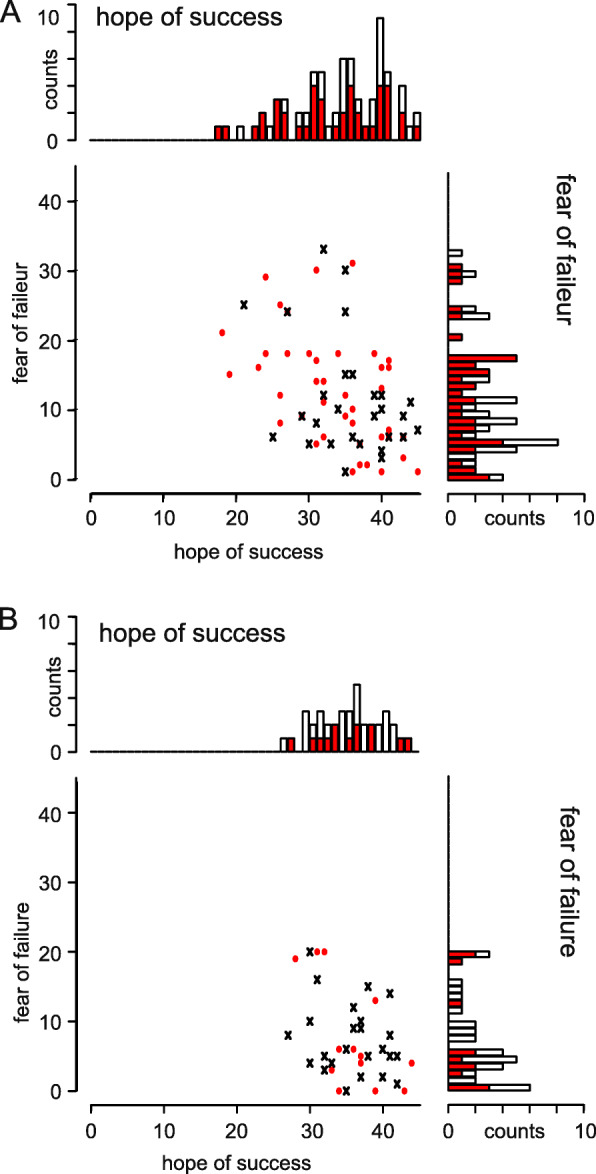
Scatterplots and stacked bar charts of the scores *hope of success* and *fear of failure* of amateur athletes. **a** female players and **b** male players. For the scatterplot: red dots indicate the individual data of OTC analgesic users and black crosses indicate the individual data of non-users; for the stacked bar charts: red filled bars indicate the frequencies of OTC analgesic users and black open bars show the frequencies of non-users, binwidth for all bar charts: 1

In total, 108 of the 114 volleyball players filled out the questionnaire AMS-Sport. The median HS score was 36 with an interquartile range (IQR) of 9 and a range of 18–45, indicating a mild skewed distribution towards higher values. The median FF score was 9 with an IQR of 10 and a range of 0–33, indicating a moderate skewed distribution. FF was dependent on the sex of the players (Fig. 3a,b); statistically significantly higher FF values were found for women (median = 10, IQR = 11, range: 1–33, *N* = 70) than men (median = 5.5, IQR = 7, range: 0–20, *N* = 38; Wilcoxon rank-sum test W = 1813.5, *p* < 0.002). Pairwise testing between the two sexes and the two subgroups “OTC analgesic user” and “non-user” showed that this significance was driven by the difference of all male subgroups against all female subgroups, at least on a trend level (*p* < 0.1 with false-discovery-rate adjustment). Regarding sex dependency of HS (Fig. 3a,b), no difference was observed (women: median = 35, IQR = 9, range: 18–45, *N* = 70 versus men: median = 36, IQR = 7, range: 27–44, *N* = 38; Wilcoxon rank-sum test W = 1195.5, *p* = 0.388). Looking at the *net hope* (NH, Table 5), a significant sex difference could be seen (Wilcoxon rank-sum test, W = 951.5, *p* = 0.015). Pairwise testing showed that this was due to the difference between the subgroups “female OTC analgesic user” versus “male OTC analgesic non-user” (*p* < 0.057 with false-discovery-rate adjustment). There was no significant difference within the sexes (Table 5).

**Table 5:** AMS-Sport scores hope of success (HS), fear of failure (FF), and net hope (NH) of amateur athletes

	Score	OTC analgesics-user	non-user
women	HS	33.5 ± 6.8, 18–45, 41	35.8 ± 5.8, 21–45, 29
	FF	12.6 ± 8.1, 1–31, 41	11.2 ± 8.3, 1–33, 29
	NH	20.9 ± 12.9, −5–44, 41	24.5 ± 11.8, −4–38, 29
men	HS	35.9 ± 4.6, 28–44, 13	35.8 ± 4.3, 27–42, 25
	FF	7.7 ± 7.6, 0–20, 13	7.2 ± 5.2, 0–20, 25
	NH	28.2 ± 11.1, 9–43, 13	28.7 ± 7.4, 10–41, 25

The data are separated by sex and use of OTC analgesics. Values are presented as mean ± SD, range, *N*

### Logistic regression modelling

**Fig. 4:**
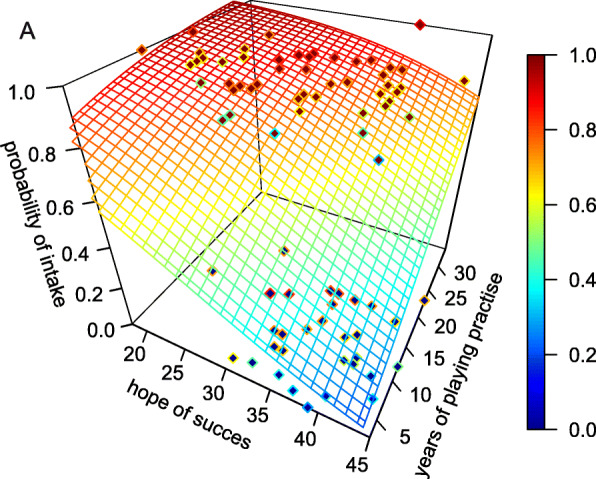
Probability of the use of OTC analgesics in amateur female players. The probability is given as a function of the scale *hope of success* and the *years of playing practise*. The x-axis shows the score *hope of success*, the y-axis shows the *years of playing practise* of the player, and the z-axis indicate the probability of taking OTC analgesics. The grid represents the logistic regression function given by the parameter *hope of success* = − 0.0867 and *years of playing practise* = 0.0697 (for details see text), the probability is colour coded (scale on the right side). Red diamond: data of female OTC analgesic users, blue diamond: data of female non-users

For estimating the influence of all parameters queried in the present study on the use of OTC analgesics, a logistic regression model of the probability of drug use was calculated for both sexes. For female players, the parameter estimation of the complete model revealed that it only yields nominal values for HS (HS: − 0.0934 ± 0.047, *p* = 0.049). Model variability was estimated using the Akaike information criterion (AIC). The AIC of the complete model was 120.2 and could be reduced to 114.3 by eliminating parameters. The remaining model revealed that the parameters HS (− 0.0867 ± 0.041, *p* = 0.037) and years of playing practise (0.0697 ± 0.043, *p* = 0.103) with an intercept of 2.5641 ± 1.550 (*p* = 0.098) were sufficient to explain the data. This means that an increase of the HS by 10 points for a given number of years of playing practise will result in a reduction of the probability of drug intake to 78% of the previous value. Regarding the athlete’s years of playing practise at a given HS, an increase of about 10 years enhanced the probability of drug intake by about 27%. The relationship between the probability of drug intake as a function of the HS score and the years of playing practise is shown in Fig. 4. For male players, the parameter estimation of the complete model revealed no relevant value (all *p*-values above 0.5). Model reduction using AIC (from 64.8 to 55.5) revealed that in men, the probability of drug intake did not depend on any of the model parameters studied.

## Discussion

This study describes the epidemiology of OTC analgesic use in German amateur volleyball players with regard to the complaints and the sports-related circumstances leading to their use. The data analysis focused on the performance motivation as an important parameter in determining the use of OTC analgesics. The results showed that the prevalence for OTC analgesic use was sex dependent, with a higher prevalence for female players (60%) versus male players (38%). The most commonly used OTC analgesic was ibuprofen, taken over a period of 5.2 years more or less continuously. Applying a logistic regression model for estimating the probability of drug use the analysis revealed that for female players the drug use can be explained by the AMS-Sport score *hope of success* and the factor *years of playing practise*. A higher HS score reduced the probability of OTC analgesic use, while a higher age increased the probability of drug use.

### Complaints

The results of this study showed a sex-dependent difference in the prevalence of complaints, which was 1.7 times higher in women than in men; this value corresponds to previously published data (mean: 1.51; 95% confidence interval, 1.19–1.90, [28]). The complaints most frequently reported by the athletes in the present study were pain in the head, the shoulder and the back. This observation is in contrast to published findings indicating ankle sprains and knee injuries to be named most often, followed by the shoulder and the back/trunk [28, 59, 64, 65]; in these studies, head/face complains were listed as the fifth (or later) most common issue [28, 59, 64, 65]. The discrepancy between the current results and those of previous studies could be due to different query methods. In this study, participants were explicitly asked about their self-medication, whereas previous studies analysed paper-based reports from coaches [64] or team physicians [59]. In addition, studies exist that consist of a specific web-based questionnaire [28, 65] conducted by a team’s coach [66] and report on athletes’ injury-related absences from sports (whereby such absences were likely ordered by a physician, physiotherapist or athletic trainer) ranging from < 1 week (61% [65];) up to > 3 weeks (13%; 65) with the longest duration reported for ankle sprain injuries with approximately 4.5 weeks [64]. Overall, it can be assumed that injury treatments reported in the literature have been carried out under the supervision of a physician or a physiotherapist or a sports coach, but not by the athlete her/himself. To target only self-medication in the present study, the data of athletes taking OTC analgesics due to a physician’s advice was excluded. This is the first study that analysed amateur athletes’ self-medication that was not covered by medical or other care. This may lead to different results compared to those of previous studies. In the present study participants reported complaints leading to the use of pain medication (and no other complaints or injuries) and due to anonymous reporting, both, sports-related and non-sports-related complaints may be reported.

The high prevalence of head pain observed in the present study compared to previous studies is striking. Headaches are one of the most prevalent neurological disorders and can occur during a wide range of lifespan with different prevalence for both sexes [67]. Sex differences have been shown for the three most common forms of headache in Germany [68] with following values for tension-type headache (TTH): ♀ = 19.3%, ♂ = 17.1%, probable tension-type headache: ♀ = 11.4%, ♂ = 9.7%%, and migraine: ♀ = 10.6%, ♂ = 2.5%. The 6-month prevalence of headache in female players is 26.3% and significant higher than the prevalence of TTH, the most frequently mentioned type of headache in Germany (18.9–20.9% for both sexes [68]). A possible cause of headache might be a concussion, most often caused by contact between a player’s head and the ball [28]. Studies have shown that concussions have an overall injury rate of 5–9% [24, 28, 65]. Concussion has long been recognized as a serious disease in American sports (for review see: [69–72]). In the analysis of US-national college volleyball players from 2013 to 2015, concussions were the second most common cause of time lost until the next training session or competition (19 and 15% for men and women, respectively [28];). The German Federal Institute of Sports Science only started issuing guidelines on concussions in 2015 [73]. Therefore, the prevalence of headaches found in this study may be a consequence of unrecognized concussions self-treated with OTC analgesics. Moreover, the higher prevalence of headaches and conditions associated with headaches (e.g. concussions) in women compared to men might be explained by the sex dependent differences in playing behaviour on the one hand and biomechanics on the other hand. The maximum ball speed during spiking is about 24.5 m/s for men and 17.9 m/s for women (for review see: [74]). Since the ball is equally heavy for both sexes, the impulse of the ball for women is about 73% of the impulse for men. Yet, the maximum strength of the head and neck muscles in women is only 64% of the strength of those muscles in men [75–78]; hence, women are not as well equipped to withstand the impact of a ball and, thus, may more likely suffer from concussions than men.

Elite athletes often do not consider severe headaches to be a reason to withdraw from competition [79]. There are two groups based on their willingness to compete while hurt. The first group consists of athletes who are conditionally willing to rest and tend to follow the recommendations of their coaches, physicians and physiotherapists and the second group consists of the rest-averse and pain-trivializing athletes who refuse to miss any competition because of severe pain or an illness that can be treated by taking painkillers or antibiotics [79]. This rest-averse and pain-trivializing group tends to include athletes who face high intrinsic pressures. The athletes in the present study had high NH score. Thus, these athletes likely belong to the pain-trivializing group taking performance situations as a challenge and being convinced to achieve the goals. Importantly, behaviour of pain-trivializing athletes, e.g., fuelled by discussions about the use of OTC analgesics, are reinforced by direct and indirect social pressure [79].

### Use of over-the-counter analgesics

In the present study, 60% of the female volleyball players and only 38% of the male players stated that they use OTC analgesics during sport exercises to compensate for sports related pain. There is evidence that women and men experience and report pain differently. Sex differences of experimentally induced pain have been described in the literature, reporting that women perceive physically or chemically induced pain more strongly than men [49–54, 80, 81]. These differences are not restricted to tactile perception and can be supposed for the whole body [82–84]. Regarding sex differences in clinical pain there are studies analysing pre-operative pain; a sex difference was found with greater pain scores for females than males [85–89], which was still detectable on the first post-operative day [86]. In terms of post-operative pain published data are inconsistent and depend partly on the time of the post-operative pain interviews [86, 87, 89]. Age and pre-operative pain influences sex-differences in post-operative pain as confounding factors [87]. Hence, one explanation for our finding that female volleyball players reported a higher consumption of OTC analgesics than male players might be that females have lower pain thresholds than males in experimental pain situations as well as greater pain scores on clinical scales. In addition, cognitive and social factors may partly explain sex-related differences in the consumption of OTC analgesics. In women 28% of the total pain perception can be explained by the fear of pain [55]. Women tend to cope better with pain when they employ pain attentional focus strategies or strategies for reinterpreting pain sensation, whereas men cope better with pain through distraction strategies [34]. Furthermore, discomfort and pain during the menstrual cycle and menstruation affect sport performance in females [90] and this is counteracted by OTC analgesics [90, 91]. Finally, past individual history may also influence female pain responses [34].

Permanent use of NSAIDs may lead to adverse drug reactions, for example, in the gastrointestinal tract, which are among the most frequent reasons for drug-related hospital admissions in Germany [92, 93]. In the present study the most frequently used OTC analgesics were ibuprofen and paracetamol, two drugs that are also taken by the German population [2]. The median of ibuprofen intake over a week was around 7% of the maximum weekly dose allowed for self-medication in Germany (range: 1–33%). Yet, the mean dose of ibuprofen taken by the athletes in our study was about 490 mg, with an individual dose per tablet varying between 200 mg and 800 mg. This is remarkable, as due to legal restrictions in Germany, packages containing tablets over 400 mg are available only by prescription. Thus, it is likely that the OTC analgesics used by the athletes had been prescribed by a physician for previous injuries and then were continued to be taken by self-medication, albeit incorrect reporting of the study participants cannot be ruled out. The recommended weekly dose of 8400 mg was not exceeded by any volleyball player participating in the present study, however, compared to the German adult population, the prevalence for drug intake was higher [2, 94]. Published data show that a dose of 400 mg ibuprofen is effective to cause moderate pain reduction without additional adverse effects (risk ratio: 0.9), but with a limited success rate (45%, measured as: > 50% pain relief over four to 6 h) [21]. Hence, the athletes’ prolonged use of ibuprofen might be due to the drug’s limited success rate. The average duration of OTC analgesic use by the athletes in our study was above the average duration of drug use in the German population independent of sex [2].

### Achievement motivation scale scores

The FF score is a direct measure of whether an athlete is motivated by the fear of failure, meaning that the athlete does not believe in success and sees him/herself as insufficiently prepared [37]. Athletes participating in the present study had higher hope scores (HS) than FF scores, resulting in positive *net hope* scores (NH), which indicates that they are confident to succeed during sporting performance situations. On average, for both sexes HS is higher than the reference group of non-elite athletes described by Wenhold et al. [37] and equally to the values of elite athletes whereas FF is comparably high as for non-elite athletes [37]. Female players had lower NH scores, and the lowest NH scores were found in the group of “female OTC analgesic user” in the present study. Therefore, one may assume that these athletes use OTC analgesics as a risk-defusing operator (RDO) [48] in the context of the use of OTC analgesics as part of a “culture of risk” behaviour among amateur athletes. Therefore, the athlete’s pain-reducing behaviour should not only be considered from a physiological or sports medicine point of view, but also in relation to the social conditions in amateur sports which should be investigated in the future.

In general, athletes take OTC analgesics in hopes of reducing the intensity of pain due to injury, illness or general complaints during exercise. However, NSAIDs have no ergogenic effects [3, 95], and poorly cured injuries may lead to further injuries, as the warning pain signal has been suppressed by the effect of the drug [24, 59, 64]. A worrying finding from the present study is the observation that 67% of participants reported taking multiple NSAIDs, especially during competition when the body was physically stressed; this leads to a further increase in the high risk of adverse effects.

### Limitations

The present study is an epidemiological study with possible selectivity of the study population. Younger athletes are more likely to fill in online questionnaires than older ones, thus, the results cannot be extrapolated to the general sports population. A potential “social desirability bias” exists, as all data were self-reported. As a methodological limitation, it can therefore not be excluded that non-sport-related complaints were also listed. As in any epidemiological study, the question of the selectivity of the study population arises. Limitations of this study result from the fact that the survey tool was a voluntary online questionnaire. For example, older respondents may fill in online questionnaires less often than younger ones. Therefore, this study is limited by a convenience sample and the results cannot be generalised to the general public. In addition, the study is limited to amateur athletes, as the number of professional athletes was too small to ensure anonymous reporting. Another limitation that skewed the sample was an overrepresentation of female respondents. The German Olympic Sports Federation states that in 2019, 196,309 men and 209,152 women were playing volleyball in Germany (age range 6 to over 60 years), corresponding to a sex ratio of men to women of 0.94. Yet, the sex ratio in this study was significantly lower, at 0.52. This difference may be a consequence of different coping strategies (see above). Women more likely replied to the questionnaire as a pain attentional focus strategy, whereas men replied to the survey as a pain distractor strategy. The actual definition and frequency of complaints that the participants cited as the reason for using OTC analgesics were only self-reported and not professionally documented. This may lead to a recall bias and can affect the reliability of the record of the dosage and frequency of taking OTC analgesics.

## Conclusion

The average duration of taking OTC analgesics was higher in volleyball players than in the German population, and the prevalence of OTC analgesic use was higher in female compared to male players. For reducing the prevalence, sport teams should implement education programs about the risks and adverse effects of OTC analgesic use. In addition, sex-specific sport psychological support should be offered to reduce the athletes` fear of failure and the direct and indirect social pressures the athletes are faced with. The use of a pain diary for self-monitoring and self-reflection might be considered. This could reduce recall biases regarding the reasons and the amount of OTC analgesics taken In this way, as a countermeasure to the “culture of risk”, a “culture of precaution” could be introduced to support healthy and positive lifelong participation in sport [40].

## Supplementary Information

**Additional file 1:.** Questionnaire in German and English.

## Data Availability

The datasets generated and analysed during the current study are not publicly available due to local legal restriction but are available from the corresponding author on reasonable request.

## Abbreviations

AIC: Akaike information criterion
IQR: Inter-quartile range
FF: *Fear of failure* score
HS: *Hope of success* score
NH: *Net hope* score
NSAIDs: Non-steroidal anti-inflammatory drugs
OTC analgesic: Over-the-counter analgesic
RDO: Risk-defusing operator
RMDT: Risk management decision theory
TTH: Tension-type headache
