# Supplementary material for: Sex differences in the consumption of over-the-counter analgesics among amateur volleyball players
Source: BMC Sports Sci Med Rehabil. 2021 Apr 29;13:45. doi: 10.1186/s13102-021-00273-5 (PMC8082781; doi:10.1186/s13102-021-00273-5)
Supplement: Supplementary file 1 — Additional file 1:. Questionnaire in German and English. [file 13102_2021_273_MOESM1_ESM.pdf]

Supplement to

Sex differences in the consumption of over-the-counter analgesics among amateur volleyball players

by Lisa Hager, Beate Averbeck, Claudia Voelcker-Rehage, Dieter F. Kutz

Questionnaire in German and English

Note: All amateur players used the German version. Both forms of the questionnaire (German and English) contains the Achievement Motives Scale - Sport (AMS-Sport) by Wenhold et al (2009). The use of the AMS is restricted by copyright.

Wenhold F, Meier C, Elbe A-M, Beckmann J. Achievement Motives Scale - Sport (AMS-Sport): Fragebogen zum Leistungsmotiv im Sport. 1. Auflage. Bundesinstitut für Sportwissenschaft; 2009.

FRAGEBOGEN Zur Ermittlung der Einnahme von rezeptfreien Schmerzmitteln im Volleyball

Es werden im folgendem Fragebogen ausschließlich Schmerzmittel betrachtet, welche oral eingenommen werden. (Tabletten, Kapseln, Tropfen, Gel)

Hallo lieber Teilnehmer,

vielen Dank, dass Sie sich Zeit nehmen, an dieser Befragung teilzunehmen.

Bei Fragen oder Problemen können Sie sich an folgende Personen wenden:

PD Dr. Dieter F. Kutz	Thüringer Weg 11, 09126 Chemnitz<br>dieter.kutz@hsw.tu-chemnitz.de
Lisa Hager (BA. Sc.)	lisa.hager@s2013.tu-chemnitz.de

Wir weisen darauf hin, dass dieses Programm aus technischen Gründen Cookies verwendet.

Diese Umfrage enthält 30 Fragen.

Probandeninformation

[]

Schmerzmittelkonsum nichtrezeptpflichtiger Analgetika im Mannschaftssport am Beispiel Volleyball Probandeninformation

a) Einleitung
Im Sport ist ein Trend zur Einnahme von nicht als Dopingmittel gelisteten Medikamenten zur Trainingsunterstützung zu verzeichnen. Von Interesse ist die Häufigkeit des Schmerzmittelkonsums in Hinblick auf einen definierten Zeitraum von sechs Monaten. Hierzu zählen nicht-rezeptpflichtige Schmerzmittel (z.B. Aspirin/ASS, Ibuprofen, Diclofenac etc.).
b) Zielsetzung der Studie
Die Untersuchung soll helfen Häufigkeit / Menge der Einnahme rezeptfreier Schmerzmittel in der Mannschaftssportart Volleyball zu bestimmen.
c) Untersuchungsablauf
Sie werden einen online-Fragebogen ausfüllen.
Dauer der Untersuchung: Die Gesamtdauer der Messung beträgt etwa 15 Minuten.
d) Vor- und Nachteile für ProbandenInnen / Risiko:
Nachteile: Zeitlicher Aufwand: 15 Minuten.
Mit der Messung sind keine erkennbaren Risiken verbunden.
e) Ein- und Ausschlusskriterien
Es werden Spielerinnen oder Spieler eines in der Bundesrepublik Deutschland ansässigen Sportvereins untersucht.
f) Art der Finanzierung des Projektes:
Das Projekt wird durch Mittel des Institutes für Angewandte Bewegungswissenschaften der TU Chemnitz finanziert.
g) Angaben über Art und Ausmaß einer allfälligen Entschädigung:
Es wird keine Aufwandsentschädigung geleistet.
h) Hinweis auf Rücktrittsrecht:
Es ist gewährleistet, dass Sie sich jederzeit ohne Angaben von Gründen und ohne, dass ein Nachteil für Sie entsteht, vom Test zurückziehen können.
i) Hinweis auf Datenschutz:
Alle persönlichen Informationen werden streng vertraulich behandelt. Nur an der Studie direkt beteiligte Mitarbeiter (namentlich Lisa Hager, PD Dr. Kutz) haben Einsicht in die persönlichen Daten. Diese werden ausschließlich für statistische Analysen und für wissenschaftliche Zwecke in anonymisierter Form weiterverwendet. Das bedeutet, dass die Daten nicht re-identifiziert werden können.
j) Angabe über Kontaktpersonen bei Fragen und beim Auftreten von Schwierigkeiten:
Bei Fragen oder auftretenden Schwierigkeiten können Sie jederzeit Kontakt mit Frau Hager aufnehmen: lisa.hager@s2013.tu-chemnitz.de *

Bitte wählen Sie alle zutreffenden Antworten aus:

☐ Ich habe die Probandenaufklärung aufmerksam gelesen und verstanden.

Einwilligungserklärung

[]

Einwilligungserklärung

Hiermit erkläre ich, dass ich insbesondere über die Ziele, den Ablauf samt Dauer und den Nutzen der Studie aufgeklärt wurde.

Die Probandeninformation habe ich gelesen und verstanden. Ich bin damit auch über den Zweck, den Ablauf, die Bedeutung sowie die Vorteile und Risiken, die damit verbunden sein können, ausführlich und verständlich aufgeklärt worden. Alle meine Fragen sind zu meiner Zufriedenheit beantwortet worden. Ich hatte genügend Zeit, um meine Entscheidung zur Studienteilnahme zu überdenken und frei zu treffen.
Mir ist bekannt, dass ich jederzeit und ohne Angabe von Gründen meine Einwilligung zur Teilnahme an der Studie zurückziehen kann, ohne dass mir daraus Nachteile entstehen.

Hinweis:
Die erhobenen Daten können nach Beendigung der Befragung aufgrund der Anonymität nachwirkend nicht mehr gelöscht werden. Somit entfällt das Widerrufsrecht für den Probanden.

Datenschutzrechtliche Einwilligungserklärung
Mir ist bekannt, dass bei der Studie personenbezogene Daten von mir erhoben, gespeichert und ausgewertet werden sollen. Ich bin damit einverstanden, dass der Projektleiter sowie mit der Studie betraute Mitarbeiter Einblick in meine personenbezogenen Daten nehmen. Ich stimme zu, dass Daten, die meine Person betreffen, unter der Verantwortung des Projektleiters in verschlüsselter Form für Studien mit einer wissenschaftlich in Betracht kommenden Fragestellung gespeichert und verarbeitet werden. Die Einwilligung zur Erhebung und zur Verarbeitung meiner personenbezogenen Daten (insbesondere der Angaben über meine Gesundheit) ist unwiderruflich. Ich bin aber bereits darüber aufgeklärt worden, dass ich jederzeit die Teilnahme an der Studie beenden kann. Ich bin mir bewusst, dass aufgrund der anonymisierten Speicherung meiner Daten deren Löschung auf meinen Wunsch hin nicht möglich ist. Unabhängig davon müssen alle Stellen, die meine personenbezogenen Daten gespeichert haben, unverzüglich prüfen, inwieweit die gespeicherten Daten noch erforderlich sind, falls ich meine Einwilligung, an der Studie teilzunehmen, widerrufe. Nicht mehr benötigte Daten sind unverzüglich zu löschen.
Die Einhaltung der datenschutzrechtlichen Vorschriften wurde mir ausdrücklich zugesichert, insbesondere des Sondertatbestandes des § 36 SächsDSG zur Verarbeitung personenbezogener Daten zu Zwecken der wissenschaftlichen Forschung.

Die Weitergabe der erhobenen Daten an Dritte (das heißt Personen, die nicht an der Technischen Universität Chemnitz zur Geheimhaltung verpflichtet sind), die Auswertung sowie die Veröffentlichung der Daten erfolgt ausschließlich in anonymisierter Form (das heißt, dass ein Personenbezug allein anhand dieser Daten nicht hergestellt werden kann). *****

Bitte wählen Sie alle zutreffenden Antworten aus:

☐ Ich erkläre mich bereit, an der o. g. Studie freiwillig teilzunehmen. Mir ist bewusst, dass ich nach dem Abschicken des Fragebogens nicht mehr die Möglichkeit des Widerrufs habe.

Fragen zur ärztlichen Verordnung

[]Sind Sie zum gegewärtigen Zeitpunkt wegen Schmerzen in ärztlicher Behandlung?

Bitte wählen Sie nur eine der folgenden Antworten aus:

- Ja
- Nein

[]Hat Ihnen der Arzt Schmerzmittel verschrieben?

Beantworten Sie diese Frage nur, wenn folgende Bedingungen erfüllt sind:
Antwort war 'Ja' bei Frage '3 [A1]' (Sind Sie zum gegewärtigen Zeitpunkt wegen Schmerzen in ärztlicher Behandlung?)

Bitte wählen Sie nur eine der folgenden Antworten aus:

- Ja
- Nein

[]Welche Schmerzmittel wurden verschrieben?

Beantworten Sie diese Frage nur, wenn folgende Bedingungen erfüllt sind:
Antwort war 'Ja' bei Frage '4 [A2]' (Hat Ihnen der Arzt Schmerzmittel verschrieben?)

Bitte geben Sie Ihre Antwort hier ein:

[]Aufgrund welcher Beschwerden nehmen Sie diese Schmerzmittel ein?

Beantworten Sie diese Frage nur, wenn folgende Bedingungen erfüllt sind:
Antwort war 'Ja' bei Frage '4 [A2]' (Hat Ihnen der Arzt Schmerzmittel verschrieben?)

Bitte geben Sie Ihre Antwort hier ein:

Fragen zum rezeptfreien Schmerzmittelkonsum

[]Nehmen Sie eigenständig und ohne ärztliche Kontrolle Schmerzmittel ein?

Bitte wählen Sie nur eine der folgenden Antworten aus:

- Ja
- Nein

[]

Wie lang nehmen Sie Schmerzmittel ohne ärztliche Aufsicht ein? (in Jahre)

Beantworten Sie diese Frage nur, wenn folgende Bedingungen erfüllt sind:
Antwort war 'Ja' bei Frage '7 [B1]' (Nehmen Sie eigenständig und ohne ärztliche Kontrolle Schmerzmittel ein?)

In dieses Feld dürfen nur Zahlen eingegeben werden.

Bitte geben Sie Ihre Antwort hier ein:

Auch wenn zeitliche Unterbrechungen vorhanden sind.

[]

Welche Schmerzmittel nehmen Sie ein?

Beantworten Sie diese Frage nur, wenn folgende Bedingungen erfüllt sind:
Antwort war 'Ja' bei Frage '7 [B1]' (Nehmen Sie eigenständig und ohne ärztliche Kontrolle Schmerzmittel ein?)

Bitte wählen Sie die zutreffende Antwort für jeden Punkt aus:

	Keine	Unregelmäßig	Regelmäßig
Aspirin	○	○	○
Diclofenac	○	○	○
Ibuprofen	○	○	○
Naproxen	○	○	○
Paracetamol	○	○	○

[]Nehmen Sie noch andere Schmerzmittel ein, welche nicht in der vorhergehenden Tabelle abgefragt wurden?

Beantworten Sie diese Frage nur, wenn folgende Bedingungen erfüllt sind:
Antwort war 'Ja' bei Frage '7 [B1]' (Nehmen Sie eigenständig und ohne ärztliche Kontrolle Schmerzmittel ein?)

Bitte wählen Sie nur eine der folgenden Antworten aus:

- Ja
- Nein

[]Welche anderen Schmerzmittel nehmen Sie ein?

Beantworten Sie diese Frage nur, wenn folgende Bedingungen erfüllt sind:
Antwort war 'Ja' bei Frage '10 [B31]' (Nehmen Sie noch andere Schmerzmittel ein, welche nicht in der vorhergehenden Tabelle abgefragt wurden?)

Bitte geben Sie Ihre Antwort hier ein:

[]

Aufgrund welcher Beschwerden nehmen Sie diese Schmerzmittel ein?

Beantworten Sie diese Frage nur, wenn folgende Bedingungen erfüllt sind:
Antwort war 'Ja' bei Frage '7 [B1]' (Nehmen Sie eigenständig und ohne ärztliche Kontrolle Schmerzmittel ein?)

Bitte geben Sie Ihre Antwort hier ein:

[]

In welchen Situationen nehmen Sie Schmerzmittel ein?

Beantworten Sie diese Frage nur, wenn folgende Bedingungen erfüllt sind:
Antwort war 'Ja' bei Frage '7 [B1]' (Nehmen Sie eigenständig und ohne ärztliche Kontrolle Schmerzmittel ein?)

Bitte wählen Sie die zutreffende Antwort für jeden Punkt aus:

	Trifft gar nicht zu	Trifft eher nicht zu	Trifft eher zu	Trifft genau zu
Training	○	○	○	○
Wettkämpfe (Turniere, Punktspiele, usw.)	○	○	○	○
Freundschaftsspiele	○	○	○	○

[]Gibt es noch andere sportartbedingte Situationen in denen Sie Schmerzmittel einnehmen, welche nicht in der vorhergehenden Tabelle abgefragt wurden?

Beantworten Sie diese Frage nur, wenn folgende Bedingungen erfüllt sind:
Antwort war 'Ja' bei Frage '7 [B1]' (Nehmen Sie eigenständig und ohne ärztliche Kontrolle Schmerzmittel ein?)

Bitte wählen Sie nur eine der folgenden Antworten aus:

- ○ Ja
- ○ Nein

[]In welchen anderen sportlichen Situationen nehmen sie Schmerzmittel ein?

Beantworten Sie diese Frage nur, wenn folgende Bedingungen erfüllt sind:
Antwort war 'Ja' bei Frage '14 [B51]' (Gibt es noch andere sportartbedingte Situationen in denen Sie Schmerzmittel einnehmen, welche nicht in der vorhergehenden Tabelle abgefragt wurden?)

Bitte geben Sie Ihre Antwort hier ein:

[]Wie oft nehmen Sie in einer Woche Schmerzmittel ein? (Verpackungsname und Anzahl)

Beantworten Sie diese Frage nur, wenn folgende Bedingungen erfüllt sind:
Antwort war 'Ja' bei Frage '7 [B1]' (Nehmen Sie eigenständig und ohne ärztliche Kontrolle Schmerzmittel ein?)

Bitte geben Sie Ihre Antwort hier ein:

(Bsp: Ibuflam 500mg; 2 Tabletten)

[]Hat sich Ihr Schmerzmittelkonsum in den letzten 6 Monaten erhöht?

Beantworten Sie diese Frage nur, wenn folgende Bedingungen erfüllt sind:
Antwort war 'Ja' bei Frage '7 [B1]' (Nehmen Sie eigenständig und ohne ärztliche Kontrolle Schmerzmittel ein?)

Bitte wählen Sie nur eine der folgenden Antworten aus:

- Ja
- Nein

[]

Beantworten Sie diese Frage nur, wenn folgende Bedingungen erfüllt sind:
Antwort war 'Ja' bei Frage '17 [B7]' (Hat sich Ihr Schmerzmittelkonsum in den letzten 6 Monaten erhöht?)

Bitte geben Sie Ihre Antwort(en) hier ein:

Um wieviel?

Grund der Erhöhung

[]Reden Sie mit anderen Spielern über die Schmerzmitteleinnahme ohne ärztliche Betreuung?

Beantworten Sie diese Frage nur, wenn folgende Bedingungen erfüllt sind:
Antwort war 'Ja' bei Frage '7 [B1]' (Nehmen Sie eigenständig und ohne ärztliche Kontrolle Schmerzmittel ein?)

Bitte wählen Sie nur eine der folgenden Antworten aus:

- ○ Ja
- ○ Nein

Fragen zur Motivation

[]

Bei diesen Fragen gibt es keine richtigen oder falschen Antworten. Es geht vielmehr darum, ob eine Aussage auf Sie zutrifft oder nicht. Jeder wird die Fragen deshalb anders beantworten, und zwar so, wie es auf ihn ganz persönlich passt. Die Fragen bestehen aus einer Reihe von Sätzen (oder Feststellungen). Sie sollen jedes Mal entscheiden, inwieweit dieser Satz auf Sie zutrifft und die entsprechende Antwort ankreuzen. Die Antworten liegen zwischen "trifft genau auf mich zu" und "trifft auf mich überhaupt nicht zu.

Bitte überlegen Sie nicht lange, sondern geben Sie die erste spontane Antwort, die Ihnen in den Sinn kommt!

Bitte wählen Sie die zutreffende Antwort für jeden Punkt aus:

	Trifft genau auf mich zu	Trifft überwiegend (größtenteils) auf mich zu	Trifft weniger (nur teilweise) auf mich zu	Trifft auf mich überhaupt nicht zu
1. Ich merke, dass mein Interesse schnell erwacht, wenn ich vor einer sportlichen Herausforderung stehe, die ich nicht auf Anhieb schaffe.	○	○	○	○
2. Wenn mir im Sport eine Herausforderung gestellt wird, die ich möglicherweise lösen kann, dann reizt es mich, diese sofort in Angriff zu nehmen.	○	○	○	○
3. Bei dem, was ich im Sport mache, will ich erfolgreich sein.	○	○	○	○
4. Mich reizen sportliche Herausforderungen, in denen ich die Möglichkeit habe, meine Fähigkeiten zu prüfen.	○	○	○	○
5. Mir gefallen sportliche Herausforderungen, von denen ich nicht genau weiß, ob ich sie auch schaffe.	○	○	○	○
6. Wenn mir eine sportliche Aufgabe gestellt wird, von der ich nicht weiß, ob ich sie wirklich bewältigen kann, reizt es mich, sofort loszulegen.	○	○	○	○

	Trifft genau auf mich zu	Trifft überwiegend (größtenteils) auf mich zu	Trifft weniger (nur teilweise) auf mich zu	Trifft auf mich überhaupt nicht zu
7. Sportsituationen, in denen ich von meinen Fähigkeiten Gebrauch machen kann, machen mir Spaß.	○	○	○	○
8. Ich mag es, vor eine etwas schwierige sportliche Aufgabe gestellt werden.	○	○	○	○
9. Ich mag Situationen im Sport, in denen ich feststellen kann, wie gut ich bin	○	○	○	○
10. Mir gefällt es im Sport, etwas Neues und Unbekanntes auszuprobieren, auch wenn es nicht gleich klappt.	○	○	○	○
11. Es ist mir wichtig, sportliche Aufgaben, die ich mir zutraue, auch tatsächlich zu schaffen.	○	○	○	○
12. Mich reizen Sportsituationen, in denen ich meine Fähigkeiten testen kann.	○	○	○	○
13. Es macht mir Spaß, mich in sportlichen Aufgaben zu engagieren, die für mich ein bisschen schwierig sind.	○	○	○	○
14. Sportliche Aufgaben, die etwas schwierig zu bewältigen sind, reizen mich.	○	○	○	○
15. Mir gefällt es, im Sport etwas Neues zu lernen, auch wenn es nicht gerade in meinen eigentlichen Sportbereich fällt	○	○	○	○

aktuelle Fassung des Fragebogens (AMS-Sport)
Wenhold, F., Elbe, A.-M. & Beckmann, J. (2008).*AMS-Sport Langversion: Allgemeiner Fragebogen zum Leistungsmotiv im Sport*. Abgerufen aus dem World Wide Web am 26.08.2018 unter www.bisp.de.

Fragen zur Motivation 2

[]

Bei diesen Fragen gibt es keine richtigen oder falschen Antworten. Es geht vielmehr darum, ob eine Aussage auf Sie zutrifft oder nicht. Jeder wird die Fragen deshalb anders beantworten, und zwar so, wie es auf ihn ganz persönlich passt. Die Fragen bestehen aus einer Reihe von Sätzen (oder Feststellungen). Sie sollen jedes Mal entscheiden, inwieweit dieser Satz auf Sie zutrifft und die entsprechende Antwort ankreuzen. Die Antworten liegen zwischen "trifft genau auf mich zu" und "trifft auf mich überhaupt nicht zu.

Bitte überlegen Sie nicht lange, sondern geben Sie die erste spontane Antwort, die Ihnen in den Sinn kommt!

Bitte wählen Sie die zutreffende Antwort für jeden Punkt aus:

	Trifft genau auf mich zu	Trifft überwiegend (größtenteils) auf mich zu	Trifft weniger (nur teilweise) auf mich zu	Trifft auf mich überhaupt nicht zu
16. Es gefällt mir nicht im Sport, an etwas zu arbeiten, wenn ich nicht sicher bin, dass ich es schaffe.	○	○	○	○
17. Sportsituationen, in denen meine Fähigkeiten auf die Probe gestellt werden, mag ich nicht.	○	○	○	○
18. Wenn im Sport eine Aufgabe etwas schwierig ist, hoffe ich, dass ich es nicht machen muss, weil ich Angst habe, es nicht zu schaffen.	○	○	○	○
19. Wenn ich eine sportliche Aufgabe nicht sofort schaffe, werde ich ängstlich.	○	○	○	○
20. Sportsituationen, in denen ich meine Fähigkeiten anwenden kann, gehe ich am liebsten aus dem Weg.	○	○	○	○
21. Es beunruhigt mich im Sport, etwas zu tun, wenn ich nicht sicher bin, dass ich es schaffen kann.	○	○	○	○
22. Auch wenn niemand zuguckt, fühle ich mich in neuen Sportsituationen ziemlich ängstlich	○	○	○	○
23. Sportliche Aufgaben, die ich nicht schaffen kann, machen mir Angst, auch dann, wenn niemand meinen Misserfolg bemerkt.	○	○	○	○

	Trifft genau auf mich zu	Trifft überwiegend (größtenteils) auf mich zu	Trifft weniger (nur teilweise) auf mich zu	Trifft auf mich überhaupt nicht zu
24. Auch bei sportlichen Herausforderungen, von denen ich glaube, dass ich sie kann, habe ich Angst zu versagen.	○	○	○	○
25. Wenn ich im Sport etwas nicht geschafft habe, schäme ich mich auch dann, wenn ich nicht darauf angesprochen werde.	○	○	○	○
26. In etwas schwierigen Sportsituationen, in denen viel von mir abhängt, habe ich Angst zu versagen	○	○	○	○
27. Ich finde es beunruhigend eine sportliche Tätigkeit auszuführen, bei der ich meine Fähigkeiten unter Beweis stellen muss.	○	○	○	○
28. Schon die Vorstellung im Sport vor eine neue unbekannte Herausforderung gestellt zu werden, macht mich etwas ängstlich.	○	○	○	○
29. Mir gefällt es nicht mich auf eine sportliche Aufgabe einzulassen, wenn ich nicht sicher bin, ob ich sie schaffe.	○	○	○	○
30. Leistungsanforderungen im Sport, die etwas schwierig sind, beunruhigen mich.	○	○	○	○

aktuelle Fassung des Fragebogens (AMS-Sport)
Wenhold, F., Elbe, A.-M. & Beckmann, J. (2008).*AMS-Sport Langversion: Allgemeiner Fragebogen zum Leistungsmotiv im Sport*. Abgerufen aus dem World Wide Web am 26.08.2018 unter www.bisp.de.

Allgemeine Fragen

[]Geschlecht

Bitte wählen Sie nur eine der folgenden Antworten aus:

- ○ weiblich
- ○ männlich

[]Wie alt sind Sie?

In dieses Feld dürfen nur Zahlen eingegeben werden.

Bitte geben Sie Ihre Antwort hier ein:

[ ]

[]Seit wieviel Jahren spielen Sie Volleyball?

In dieses Feld dürfen nur Zahlen eingegeben werden.

Bitte geben Sie Ihre Antwort hier ein:

[ ]

[]Wieviel Stunden Sport pro Woche treiben Sie? (inklusive Volleyball)

In dieses Feld dürfen nur Zahlen eingegeben werden.

Bitte geben Sie Ihre Antwort hier ein:

[ ]

Stunden pro Woche

[]Nehmen Sie regelmäßig an Wettkämpfen teil?

Bitte wählen Sie nur eine der folgenden Antworten aus:

- ○ Ja
- ○ Nein

[]In welcher höchsten Spielklasse spielen Sie?

Bitte wählen Sie nur eine der folgenden Antworten aus:

- Nationalmannschaft
- 1. Bundesliga
- 2. Bundesliga
- 3. Liga Nord
- 3. Liga Süd
- Regionalliga
- Bayern- /Hessen- /Sachsenliga
- Landesliga
- Bezirksliga
- Bezirksklasse
- Kreisliga/ Kreisklasse
- Sonstiges

[]

Auf welcher Position spielen Sie überwiegend?

Bitte wählen Sie nur eine der folgenden Antworten aus:

- Mittelblock
- Außenangreifer
- Diagonalspieler
- Zuspieler
- Libero
- Sonstiges

Information Schmerzmittelkonsum

[]Im Anhang gibt es die Möglichkeit, sich über Risiken der regelmäßigen Einnahme von nicht-rezeptpflichtigen Medikamenten zu informieren.

Bitte wählen Sie nur eine der folgenden Antworten aus:

- ○ Ja, ich möchte mich informieren.
- ○ Nein Danke.

[]

Risiken bei häufiger Einnahme von nichtrezeptpflichtiger Analgetika

Die nachfolgenden Informationen sind weder als Grundlage für gesundheitsbezogene Entscheidungen noch zur Selbstdiagnosen zu verwenden. Bei gesundheitlichen Beschwerden und zur individuellen Beratung konsultieren Sie bitte Ihren Arzt. Nur eine individuelle Untersuchung kann zu einer Diagnose und Therapieentscheidung führen. Nehmen Sie Medikamente nur nach Absprache mit einem Arzt oder Apotheker ein.

Die am häufigsten genutzten nichtrezeptpflichtigen Analgetika sind nicht-steroidale Antirheumatika (NSAR) wie etwa Acetylsalicylsäure (ASS) / Aspirin, Diclofenac, Ibuprofen, Naproxen und Paracetamol. NSAR sind nur zur Kurzzeitanwendung gedacht. So liegt z.B. die maximale Anwendungsdauer unter Berücksichtigung der Tageshöchstmenge von Paracetamol bei 3 Tage, ASS und Ibuprofen bei 4 Tagen. Bei Naproxen ist die Einnahme nach Anweisung des Arztes zu erfolgen.

Bei gewohnheitsmäßiger Einnahme von Schmerzmitteln kann es vor allem zu folgenden Nebenwirkungen kommen:

**- Dauerhafte Nierenschädigung mit dem Risiko eines Nierenversagens (Analgetika-Nephropathie).
Dieses Risiko ist besonders groß, wenn Sie mehrere verschiedene Schmerzmittel kombiniert einnehmen.**

**- Blutungen des Magen-Darm-Traktes, Geschwüre und Perforationen, auch mit tödlichem Ausgang.
Sie können mit oder ohne vorherige Warnsymptome bzw. schwerwiegende Ereignisse im Magen-Darm-Trakt
zu jedem Zeitpunkt auftreten.**

**- Verändertes Blutbild mit verminderter bis fehlender Blutgerinnung.
Als Folge können jede Form von Läsionen wie Muskelfaserrisse, Blutergüsse, Gelenksverletzungen als auch
Mikroläsionen des skelettmotorischen Apparates schlechter ab heilen. Der Heilungsprozess dauert länger.
Bei nahezu fehlender Blutgerinnung können diese Verletzungen tödlichen Ausgang haben.**

**- Schwerwiegende Hautreaktionen mit Rötung und Blasenbildung (exfoliative Dermatitis, Stevens-Johnson-
Syndrom und toxische epidermale Nekrolyse/Lyell-Syndrom) sind möglich.
Diese können einen tödlichen Ausgang nehmen.**

**- Paradoxale Reaktion in Form dumpf drückender Dauerkopfschmerzen.
Bei 5 – 8% aller Kopfschmerzpatienten liegt ein Medikamentenmissbrauch zu Grunde.
Frauen sind davon fünfmal häufiger betroffen als Männer.**

Generell ist zu beachten, dass eine gleichzeitige Anwendung von verschiedenen NSAR zu vermeiden ist, da sich die Nebenwirkungen gegenseitig verstärken können. Bei Einnahme von allen Arzneimitteln in Schwangerschaft und Stillzeit ist vorher der Arzt oder Apotheker um Rat zu fragen.

Weitere Informationen finden Sie im Internet unter anderem bei der Bundeszentrale für gesundheitliche Aufklärung -https://www.bzga.de/

z.B. Medikamente – Basisinformationen - https://www.bzga.de/infomaterialien/suchtvorbeugung/medikamente-basisinformationen/

Medikamente - ab wann sind sie riskant? - Ein Heft in Leichter Sprache - https://www.bzga.de/infomaterialien/suchtvorbeugung/?idx=3044

Haben Ihnen die Informationen geholfen?

Beantworten Sie diese Frage nur, wenn folgende Bedingungen erfüllt sind:
Antwort war 'Ja, ich möchte mich informieren.' bei Frage '29 [Info1]' (Im Anhang gibt es die Möglichkeit, sich über Risiken der regelmäßigen Einnahme von nicht-rezeptpflichtigen Medikamenten zu informieren.)

Bitte wählen Sie nur eine der folgenden Antworten aus:

- ○ Ja
- ○ Nein

14.05.2019 – 09:48
Übermittlung Ihres ausgefüllten Fragebogens:
Vielen Dank für die Beantwortung des Fragebogens.

QUESTIONNAIRE to determine the use of over-the-counter painkillers in volleyball

In the following questionnaire only analgesics are taken which are orally. taken (Tablets, capsules, drops, gel)

Dear participants,

Thank you for taking your time and having one of these customers.

If you have problems or questions, you can contact a following contact person:

PD Dr. Dieter F. Kutz	Thüringer Weg 11, 09126 Chemnitz<br>dieter.kutz@hsw.tu-chemnitz.de
Lisa Hager (BA Sc)	lisa.hager@s2013.tu-chemnitz.de

There are 30 questions in this survey

Education of the test persons

[]

**Consumption of over-the-counter analgesics/ painkillers in Volleyball
Education of the test persons**

a) Introduction
There is a trend in sports towards taking medication not listed as drugs. Of interest is the frequency of analgesic use over a defined period of six months. These include non-prescription painkillers (e.g. Aspirin/ASA, Ibuprofen, Diclofenac etc.).
b) Aim of the study
The aim of the study is to determine frequency and amount of intake of over-the-counter painkillers in volleyball.
c) Examination procedure
You will complete an online questionnaire.
Duration of the examination: The total duration of the measurement is about 15 minutes.
d) Advantages and disadvantages for subjects / risks:
Disadvantage: Time required: 15 minutes.
There are no identifiable risks associated with the measurement.
e) Inclusion and exclusion criteria
Players of sports clubs based in Germany are examined.
f) Type of financing of the project:
The project is funded by means of the institute.
g) Information on the nature and extent of any compensation:
There will be no expense allowance.
h) Right of withdrawal:
It is guaranteed that you can withdraw from the test at any time without giving reasons and without causing a disadvantage for you.
i) Note on privacy:
All personal information is kept strictly confidential. Only employees directly involved in the study (namely Lisa Hager, PD Dr. Kutz) have access to personal data. These are used exclusively for statistical analysis and for scientific purposes in anonymous form. This means that the data cannot be re-identified.
j) Information about contact persons for questions and difficulties:
If you have any questions or difficulties, you can always contact Ms. Hager: lisa.hager@s2013.tu-chemnitz.de. *

Please choose **all** that apply:

☐ I have carefully read and understood the above information about the study.

Informed consent form

[]

Informed consent form

I hereby declare that I have been informed in particular about the objectives, the procedure including the duration and the benefits of the study.
I read and understood the subject information. I have also been informed in detail and comprehensible about the purpose, the process, the meaning, the advantages, and risks that may be associated with it. All my questions have been answered to my satisfaction. I had sufficient of time for my decision to participate and did it independently.
I am aware that I can withdraw my consent to participate in the study at any time, without stating any reasons, and without incurring any disadvantages.

Hint:
The collected data can`t be deleted after the end of the survey because of the anonymity. Thus, subject`s right of withdrawal is revoked.

Data protection consent
I am aware that personal data are collected, stored and evaluated during the study. I agree that the Project Leader and staff entrusted with the study can view my personal information. I agree that data concerning my person (in particular health data) will be stored and processed in encrypted form for studies with a scientifically relevant question, under the responsibility of the project leader. The consent to the collection and processing of my personal data (in particular the information about my health) is irrevocable. However, I have already been informed that I can stop participating in the study at any time. In the event of a withdrawal of my consent to participate, I agree that the data stored up to that time may continue to be used where necessary. In particular, I agree that my data will remain stored for control purposes.

I am aware that due to the anonymous storage of my data their deletion is not possible at my request. Regardless, all sites that have stored my personal information (in particular health information) must immediately assess the extent to which the stored information is still required if I revoke my consent to participate in the study. No longer required data must be deleted immediately.
I was expressly assured of compliance with the data protection regulations, in particular the special status of the Data Protection Act of Saxony § 36 SächsDSG for the processing of personal data for the purposes of scientific research.
The transfer of the collected data to third parties (i.e. persons who are not obliged to maintain secrecy at the Chemnitz University of Technology), the evaluation and the publication of the data takes place exclusively in an anonymous form (that means that a personal reference cannot be produced on the basis of these data alone). *

Please choose **all** that apply:

☐ I agree to participate for the study. I am aware that after sending the questionnaire I no longer have the option of cancelling it.

Questions about medical care

[]Are you at the moment in medical treatment due to pain?

Please choose **only one** of the following:

- Yes
- No

[]Has the doctor prescribed you painkillers?

Only answer this question if the following conditions are met:
Answer was 'Yes' at question '3 [A1]' (Are you at the moment in medical treatment due to pain?)

Please choose **only one** of the following:

- Yes
- No

[]Which painkillers?

Only answer this question if the following conditions are met:
Answer was 'Yes' at question '4 [A2]' (Has the doctor prescribed you painkillers?)

Please write your answer here:

[]Against which symptoms do you take these painkillers?

Only answer this question if the following conditions are met:
Answer was 'Yes' at question '4 [A2]' (Has the doctor prescribed you painkillers?)

Please write your answer here:

Questions about over-the-counter analgesics / painkillers consumption

[]Do you take painkillers independently and without medical supervision

Please choose **only one** of the following:

- Yes
- No

[]For how many years have you been taking painkillers on your own/ without doctor`s supervision? (in years)

Only answer this question if the following conditions are met:
Answer was 'Yes' at question '7 [B1]' (Do you take painkillers independently and without medical supervision)

In dieses Feld dürfen nur Zahlen eingegeben werden.

Please write your answer here:

Even if there are time interruptions.

[]Which analgesics in tablet form do you take?

Only answer this question if the following conditions are met:
Answer was 'Yes' at question '7 [B1]' (Do you take painkillers independently and without medical supervision)

Please choose the appropriate response for each item:

	None	irregularly	regularly
Aspirin	○	○	○
Diclofenac	○	○	○
Ibuprofen	○	○	○
Naproxen	○	○	○
Paracetamol	○	○	○

[]Are you taking any other painkillers that were not inquired in the previous question?

Only answer this question if the following conditions are met:
Answer was 'Yes' at question '7 [B1]' (Do you take painkillers independently and without medical supervision)

Please choose **only one** of the following:

- Yes
- No

[]What other painkillers do you take?

Only answer this question if the following conditions are met:
Answer was 'Yes' at question '10 [B31]' (Are you taking any other painkillers that were not inquired in the previous question?)

Please write your answer here:

[]Against which symptoms do you take these painkillers?

Only answer this question if the following conditions are met:
Answer was 'Yes' at question '7 [B1]' (Do you take painkillers independently and without medical supervision)

Please write your answer here:

[]In which situation do you take these painkillers?

Only answer this question if the following conditions are met:
Answer was 'Yes' at question '7 [B1]' (Do you take painkillers independently and without medical supervision)

Please choose the appropriate response for each item:

	strongly disagree	disagree	agree	strongly agree
Training	○	○	○	○
Competition (Tournaments, League games etc.)	○	○	○	○
Friendly matches	○	○	○	○

[]Are there any other situations in which you are taking painkillers that were not queried in the last question?

Only answer this question if the following conditions are met:
Answer was 'Yes' at question '7 [B1]' (Do you take painkillers independently and without medical supervision)

Please choose **only one** of the following:

- ○ Yes
- ○ No

[]In which situations do you take painkillers?

Only answer this question if the following conditions are met:
Answer was 'Yes' at question '14 [B51]' (Are there any other situations in which you are taking painkillers that were not queried in the last question?)

Please write your answer here:

[]How much painkillers do you take in a week? (Tablet name and number of tablets, please use the German names if known)

Only answer this question if the following conditions are met:
Answer was 'Yes' at question '7 [B1]' (Do you take painkillers independently and without medical supervision)

Please write your answer here:

(E.g.: Ibuflam 500mg Retarttabletten; 2 tablets)

[]Has your consumption of painkillers increased over the last 6 months?

Only answer this question if the following conditions are met:
Answer was 'Yes' at question '7 [B1]' (Do you take painkillers independently and without medical supervision)

Please choose **only one** of the following:

- Yes
- No

[]How much?

Only answer this question if the following conditions are met:
Answer was 'Yes' at question '17 [B7]' (Has your consumption of painkillers increased over the last 6 months?)

Please write your answer(s) here:

How much

Reason of increase

[]Are you talking to other players about taking painkillers without medical supervision?

Only answer this question if the following conditions are met:
Answer was 'Yes' at question '7 [B1]' (Do you take painkillers independently and without medical supervision)

Please choose **only one** of the following:

- ○ Yes
- ○ No

Questions about motivation

[]

There are no right or wrong answers to these questions. It's more about whether or not a statement applies to you. So everyone will answer the questions differently, in a way that suits him personally. The questions consist of a series of sentences (or statements). You should decide each time to what extent this sentence applies to you and to tick the corresponding answer. The answers lie between "meets exactly" and "does not apply to me at all”.

Please do not think long! Give the first spontaneous answer that come into yours mind!

Please choose the appropriate response for each item:

	Meets exactly	Mainly (mostly) towards me	Matches less (only partially) towards me	Does not apply to me at all
1. I realize that my interest quickly awakens when I face a sporting challenge that I can`t do right off the bat.	○	○	○	○
2. If I get a challenge in sports that I might be able to solve, I'm tempted to tackle it immediately.	○	○	○	○
3. I want to be successful in what I do in sports.	○	○	○	○
4. I am motivated by athletic challenges in which I have the opportunity to test my abilities.	○	○	○	○
5. I like athletic challenges that I don't know whether I can do them.	○	○	○	○
6. When I'm asked to do a sports job that I do not know if I can really handle, it takes me to get started right away.	○	○	○	○
7. Sport situations in which I can use my abilities are fun.	○	○	○	○
8. I like to be faced with a somewhat difficult athletic task.	○	○	○	○
9. I like situations in sports where I can see how good I am.	○	○	○	○
10. In sports I like to try something new and unknown, even if it does not work out right away.	○	○	○	○

	Meets exactly	Mainly (mostly) towards me	Matches less (only partially) towards me	Does not apply to me at all
11. It is important to me to be able to do the sporting tasks that I expect.	○	○	○	○
12. I like sports situations in which I can test my skills.	○	○	○	○
13. I enjoy engaging in athletic tasks that are a bit difficult for me.	○	○	○	○
14. Sporting tasks that are a bit difficult to master irritate me.	○	○	○	○
15. I like to learn something new in sports, even if it does not fall into my actual sports field.	○	○	○	○

- English translation by D.F. Kutz and L. Hager based on the current version of the questionnaire (AMS-Sport): Wenhold, F., Elbe, A.-M. & Beckmann, J. (2008). AMS-Sport Langversion: Allgemeiner Fragebogen zum Leistungsmotiv im Sport. Accessed from the World Wide Web on August, 26th 2018 at www.bisp.de.

Questions about motivation 2

[]

There are no right or wrong answers to these questions. It's more about whether or not a statement applies to you. So everyone will answer the questions differently, in a way that suits him personally. The questions consist of a series of sentences (or statements). You should decide each time to what extent this sentence applies to you and to tick the corresponding answer. The answers lie between "meets exactly" and "does not apply to me at all".

Please do not think long! Give the first spontaneous answer that come into yours mind!

Please choose the appropriate response for each item:

	Meets exactly	Mainly (mostly) towards me	Matches less (only partially) towards me	Does not apply to me at all
16. I don`t like working in sports when I'm not sure I can do it.	○	○	○	○
17. Sport situations in which my abilities are put to the test I don`t like.	○	○	○	○
18. If a task is a bit difficult in sports, I hope that I do not have to do it because I'm afraid I will not make it.	○	○	○	○
19. If I fail to do a sports job immediately, I become anxious.	○	○	○	○
20. I like to avoid sport situations where I can apply my skills.	○	○	○	○
21. It worries me in sports to do something when I'm not sure I can do it.	○	○	○	○
22. Even if nobody is watching, I feel quite anxious in new sports situations	○	○	○	○
23. Sporting tasks that I can`t manage scare me, even when nobody notices my failure.	○	○	○	○
24. I am afraid to fail even in sporting challenges that I believe I can do.	○	○	○	○
25. 25. If I cannot achieve something in sports, I am ashamed also if I am not addressed.	○	○	○	○

	Meets exactly	Mainly (mostly) towards me	Matches less (only partially) towards me	Does not apply to me at all
26. In a bit difficult situations, in which much depends on me, I am afraid to fail.	○	○	○	○
27. I find it troubling to do a sporting activity where I have to prove my skills.	○	○	○	○
28. Being presented with a new unknown challenge in sports makes me a bit anxious.	○	○	○	○
29. I don’t like to get involved with an athletic task if I'm not sure I can do it.	○	○	○	○
30. Performance requirements in sports that are a bit difficult worry me.	○	○	○	○

- English translation by D.F. Kutz and L. Hager based on the current version of the questionnaire (AMS-Sport): Wenhold, F., Elbe, A.-M. & Beckmann, J. (2008). AMS-Sport Langversion: Allgemeiner Fragebogen zum Leistungsmotiv im Sport. Accessed from the World Wide Web on August, 26th 2018 at www.bisp.de.

General questions

[]Gender

Please choose **only one** of the following:

- ◯ Female
- ◯ Male

[]How old are you?

In dieses Feld dürfen nur Zahlen eingegeben werden.

Please write your answer here:

[]For how many years have you been playing volleyball?

In dieses Feld dürfen nur Zahlen eingegeben werden.

Please write your answer here:

[]How many hours of sports do you do per week? (including volleyball)

In dieses Feld dürfen nur Zahlen eingegeben werden.

Please write your answer here:

Hours per week

[]Do you participate regularly in competitions?

Please choose **only one** of the following:

- ◯ Yes
- ◯ No

[]In which league do you play (German National League System)?

Please choose **only one** of the following:

- Nationalmannschaft
- Liga 1.
- Liga 2
- Liga 3 north
- Liga 3 south
- Regionalliga
- Bayern-/ Hessen-/ Sachsenliga
- Landesliga
- Bezirksliga
- Bezirksklasse
- Kreisliga/ Kreisklasse
- Other

[]Which position do you play predominantly?

Please choose **only one** of the following:

- Middle Blocker
- Outside Hitter
- Weakside Hitter
- Setter
- Libero
- Other

Information Painkiller consumption

[]The appendix gives you the opportunity to find out about the risks of taking regular non-prescription medicines.

Please choose **only one** of the following:

- Yes, I would like to inform myself.
- No, thanks.

[]

Risks associated with frequent consumption of over-the-counter analgesics/ painkillers

The following information should not be used as a basis for health-related decisions or for self-diagnosis. For health complaints and for individual advice, please consult your doctor. Only an individual examination can lead to a diagnosis and therapy decision. Take medication only after consulting a doctor or pharmacist.

The most commonly used non-prescriptive analgesics are non-steroidal anti-inflammatory drugs (NSAIDs) such as aspirin, diclofenac, ibuprofen, naproxen and paracetamol. NSAIDs are for short-term use only. For example, the maximum duration of use taking into account the daily maximum amount of paracetamol is 3 days, for ASA and ibuprofen 4 days. Naproxen should be taken as directed by the doctor.

Habitual ingestion of painkillers can cause the following side effects:
- permanent kidney damage with the risk of kidney failure (analgesic nephropathy).
This risk is especially great if you take several different painkillers combined.
- bleeding of the gastrointestinal tract, ulcers and perforations, even with fatal outcome.
They may occur at any time with or without previous warning symptoms or serious events in the gastrointestinal tract.
- altered blood picture with reduced or no blood clotting.
As a result, any form of lesion, such as torn muscle fibres, haematoma, joint injuries, and micro-lesions of the skeletal motor apparatus, may be less effective.
The healing process takes longer. With almost no blood clotting, this injury can have fatal outcome.
- severe skin reactions with redness and blistering (exfoliative dermatitis, Stevens-Johnson syndrome and toxic epidermal necrolysis / Lyell syndrome). Some of them could be fatal.
- paradoxical reaction in the form of dull-pressing constant headache.
In 5 - 8% of all headache patients, a drug abuse is based.
Women are affected five times more often than men.

In general, it should be noted that concomitant use of various NSAIDs should be avoided, as the side effects may be mutually reinforcing. During pregnancy and lactation, the doctor or pharmacist should be consulted before taking any medicines.

Further information (in German language) can be found on the internet at the

Bundeszentrale für gesundheitliche Aufklärung - https://www.bzga.de/

e.g. Medikamente – Basisinformationen - https://www.bzga.de/infomaterialien/suchtvorbeugung/medikamente-basisinformationen/

Medikamente - ab wann sind sie riskant? - Ein Heft in Leichter Sprache -https://www.bzga.de/infomaterialien/suchtvorbeugung/?idx=3044

Did the information help you?

Only answer this question if the following conditions are met:
Answer was 'Yes, I would like to inform myself.' at question '29 [Info1]' (The appendix gives you the opportunity to find out about the risks of taking regular non-prescription medicines.)

Please choose **only one** of the following:

- Yes
- No

05-14-2019 – 09:48
Submit your survey.
Thank you for completing this survey.
